# Adaptive Remodeling of Achilles Tendon: A Multi-scale Computational Model

**DOI:** 10.1371/journal.pcbi.1005106

**Published:** 2016-09-29

**Authors:** Stuart R. Young, Bruce Gardiner, Arash Mehdizadeh, Jonas Rubenson, Brian Umberger, David W. Smith

**Affiliations:** 1 Faculty of Engineering, Computing and Mathematics, University of Western Australia, Crawley, Western Australia, Australia; 2 School of Engineering and Information Technology, Murdoch University, Murdoch, Western Australia, Australia; 3 Biomechanics Laboratory, Department of Kinesiology, Pennsylvania State University, University Park, Pennsylvania, United States of America; 4 School of Sport Science, Exercise and Health, University of Western Australia, Crawley, Western Australia, Australia; 5 Department of Kinesiology, University of Massachusetts, Amherst, Massachusetts, United States of America; University of Virginia, UNITED STATES

## Abstract

While it is known that musculotendon units adapt to their load environments, there is only a limited understanding of tendon adaptation *in vivo*. Here we develop a computational model of tendon remodeling based on the premise that mechanical damage and tenocyte-mediated tendon damage and repair processes modify the distribution of its collagen fiber lengths. We explain how these processes enable the tendon to geometrically adapt to its load conditions. Based on known biological processes, mechanical and strain-dependent proteolytic fiber damage are incorporated into our tendon model. Using a stochastic model of fiber repair, it is assumed that mechanically damaged fibers are repaired longer, whereas proteolytically damaged fibers are repaired shorter, relative to their pre-damage length. To study adaptation of tendon properties to applied load, our model musculotendon unit is a simplified three-component Hill-type model of the human Achilles-soleus unit. Our model results demonstrate that the geometric equilibrium state of the Achilles tendon can coincide with minimization of the total metabolic cost of muscle activation. The proposed tendon model independently predicts rates of collagen fiber turnover that are in general agreement with *in vivo* experimental measurements. While the computational model here only represents a first step in a new approach to understanding the complex process of tendon remodeling *in vivo*, given these findings, it appears likely that the proposed framework may itself provide a useful theoretical foundation for developing valuable qualitative and quantitative insights into tendon physiology and pathology.

## Author Summary

It is now widely acknowledged that tendon plays a vital role in locomotion, while experiments have revealed that tendon is much more metabolically active than previously believed. There have been increasing numbers of papers describing the responses of tenocytes to mechanical loading and speculation about the origins of tendinopathy, but to date there is currently no basic theoretical framework describing how tendon maintains tissue homeostasis consistent with the experimental data, or indeed how tendon adapts to its environmental load conditions. Based on established biological principles of tendon damage and repair, for the first time we develop a dynamic model of tendon homeostasis that is capable of adaptation. We show that for a model soleus musculotendon unit with muscle fiber length kept constant, our model tendon is ‘capable’ of dynamically adjusting itself to find a stable equilibrium tendon geometry, which coincides with minimum metabolic cost of muscle activation. This new theoretical framework for tendon homeostasis and adaptation offers the possibility of refocusing research in basic and clinical science.

## Introduction

Tendons are dense fibrous tissues that transfer tensile forces from muscles to bones. During normal daily activity, human Achilles tendon experiences high intensity cyclic loads, up to 4–8 times the body weight [1–3]. Achilles tendon stores potential strain energy as it is stretched, which is then recovered later in the gait cycle [4, 5]. This strain energy cycling reduces muscular work and improves the economy of locomotion. Furthermore, the uncoupling of tendon and muscle lengths, due to the elastic deformation of Achilles tendon, enable the muscle fibers to operate at more favorable lengths and velocities, thus improving locomotion economy even further [6].

It is apparent that there are variable tendon geometries and properties, and that tendon tissue has the capacity to adapt to its mechanical environment [7–10], but how? Here we develop a biologically plausible computational model of tendon adaptation. The basis of our model is a collagen fiber damage and repair models, which in turn are based on known biological processes. When an Achilles tendon model is incorporated into a simplified model of the Achilles-soleus unit and allowed to adapt over time with usage, we observe the capacity of model tendon to remodel towards a stable equilibrium tendon geometry, which can coincide with minimum metabolic cost of the model musculotendon unit operation. We begin by introducing the key biological processes incorporated into the model.

Tendon extracellular matrix (ECM) is primarily composed of Type I collagen (up to 86% dry mass). Collagen fibers, run mainly along the axial length of the Achilles tendon, arranged in a hierarchical structure [11, 12]. At the smallest scale, tropocollagen molecules self-assemble into microscopically visible strands of collagen fibrils [13]. Despite the small diameter of collagen fibrils, typically 100–150 nm in human adult Achilles tendon [11, 14, 15], their total lengths are believed to be much longer, and potentially extend continuously from muscle fibers to bone [16]. A bundle of collagen fibrils form primary collagen fibers [11, 17] that are then hierarchically aggregated to form primary, secondary and tertiary fascicles across the whole tendon [11, 18–20].

Tendon ECM is maintained by resident cells known as tenocytes, which mediate the synthesis and degradation of the ECM components [21]. There is compelling evidence for continuous tendon remodeling by the tenocytes [22–24]. Normally, primary collagen fibers are enclosed by a confluent sheet of tenocytes [25]. This implies that collagen fibril adaptation must normally occur via processes acting at a distance from the tenocyte itself. The tenocytes synthesize proteases and new collagen molecules that then self-assemble to repair fibrillar damage. We envisage that these physiologic processes are consistent with homeostatic and adaptive processes within tendon [26], and it is these processes that are the focus of our proposed tendon adaptation model.

**Fig 1:**
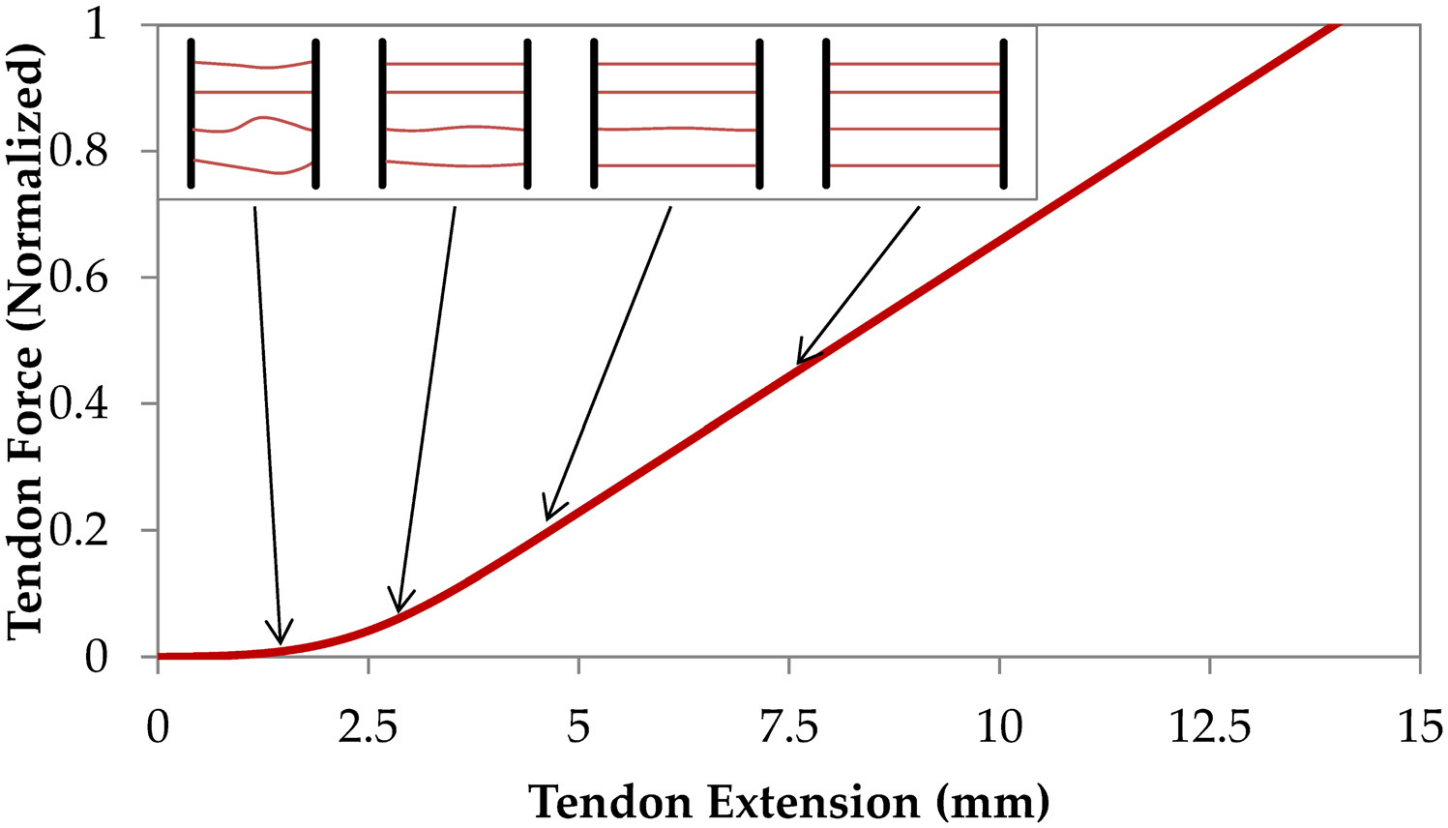
Discretized model of tendon. The mechanical response of tendon is constructed by aggregating the mechanical behavior of strings with different slack lengths, resulting in a non-linear tendon stress-strain curve.

Typically, the stress-strain response of a whole tendon is composed of four regions: (i) extension without significant force up to the tendon’s slack length, (ii) a toe region, (iii) a linear elastic region, and (iv) a failure region [27]. The slack length is defined as the tendon length at which the tendon first experiences load [28]. Most of the tendon crimp is removed during extension up to the slack length [29]. The toe region corresponds with further sequential straightening of crimped collagen fibers [29]. Experimental observations report a unique crimp pattern to each individual collagen fascicle [29–31] indicating that each fascicle's stretched length is also unique. Based on the premise of distribution of collagen fiber lengths, it is possible to reproduce tendon’s non-linear force-extension behavior [32–34], Fig 1.

Cyclic loading of Achilles tendon during habitual activities, such as walking or running, damages the tendon and initiates repair processes to maintain tendon homeostasis. It is observed that cyclic loading of tendons gradually induces ‘micro-damage‘ or ‘sub-failure injuries’ at the collagen molecular and fibrillar levels, which are collectively referred to as ‘mechanical fatigue damage’ [35–41]. The fatigue response of human Achilles tendon to cyclic mechanical loading is clearly evidenced in the experimental studies of Wren et al. [38].

At the microscale, mechanical fatigue damage to collagen fibrils may present itself in ‘focal’ or ‘generalized’ modes. Generalized damage is evidenced by repeating patterns of kinks and distortions along a number of fibrils [42], whereas focal damage is evidenced by fracture of collagen fibrils [43]. One mode of damage may dominate the other depending on the prevalence and type of cross-links within collagen fibrils [44]. More cross-linking between tropocollagen molecules results in stiffer tendons, such as Achilles and patellar tendons, and so favors the focal fatigue damage mode [44]. For simplicity, in this paper we have focused on the focal mode of damage only. Nevertheless, the damage model employed here may be modified to include other modes of collagen fiber damage as required.

In addition to mechanical damage, tenocyte-mediated proteolytic collagen degradation also occurs, and is an essential component of tendon homeostatic processes [14] as it facilitates tissue remodeling [23, 42]. In normal tendon, proteolytic damage is usually meditated primarily by members of the matrix metalloproteinases (MMPs) family of proteases [45, 46]. Intriguingly, mechanical tensile strain of collagen fibrils has been shown to reduce, and even completely prevent, proteolytic damage of collagen fibrils by collagenase MMPs at physiologically relevant strains [14, 22, 47, 48].

From the above it is clear that while the basic mechanical and physiological aspects of tendon tissue and adaptation have been studied, how these processes are integrated to produce physiologically relevant outcomes during habitual loading is not completely understood.

Here we hypothesize that through utilization of the abovementioned biological processes, Achilles tendon is capable of adaptation by remodeling its geometry, to reach a dynamic stable equilibrium that represents tendon homeostasis. In the following, we demonstrate that for a musculotendon unit with constant muscle fiber length, our proposed tendon model is able to remodel its geometry to reach a stable homeostatic equilibrium, and that this equilibrium state can coincide with minimizing the total metabolic cost of the model musculotendon unit.

## Methods

### Model Overview

In the following we first develop and then test a discretized model of Achilles tendon adaptation. The model tendon is based upon damage and repair processes that take place at the level of primary collagen fibers. The mechanical load experienced by the tendon is based upon a simplified Achilles-soleus model and experimental measurements made during normal human gait mechanics. Over time, repeated cycles of damage and repair of the collagen fibers gradually remodel the whole Achilles tendon. We note here that the timescale for the simulated sequential damage and repair processes are not critical to our proposed adaptation model, but for convenience and definiteness we have assumed a daily cycle of damage and repair. This timescale is likely to accord with at least some of the important cyclic homeostatic processes taking place within tendon [49, 50].

**Fig 2:**
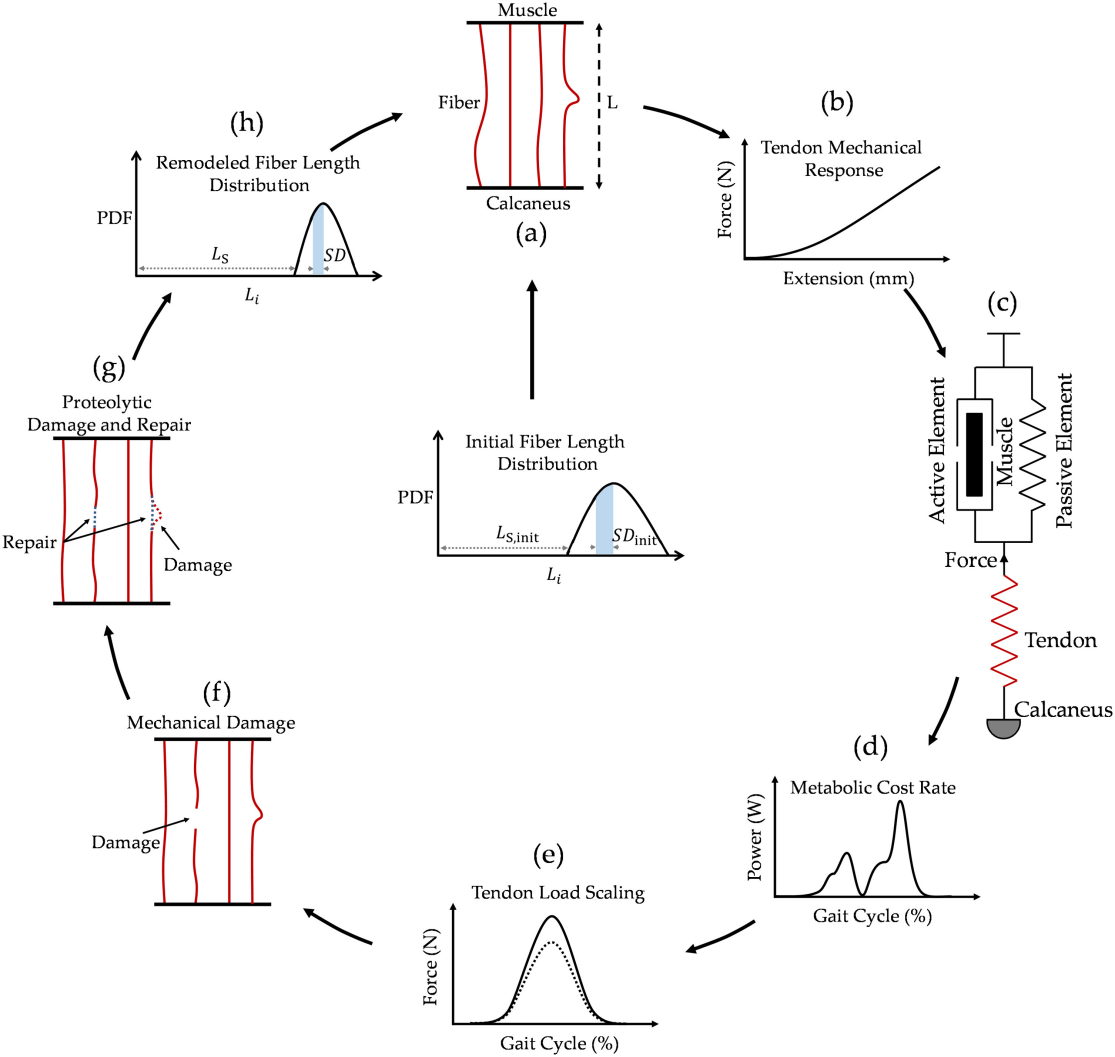
Tendon adaptation modeling cycle. a) discretized tendon model, b) force-extension response of the tendon model, c) three-component Hill musculotendon model, d) metabolic rate calculation model, e) model to scale contractile force from muscle, f) collagen fiber mechanical damage model and, g) collagen fiber proteolytic damage and repair model, h) updated tendon fiber length distribution results in a new tendon configuration and completion of a remodeling cycle of tendon adaptation model. Where PDF is probability density function, ***L***_*S*_ represents tendon slack length that is equal to the slack length of the shortest fiber, ***SD*** represents fiber length standard deviation, a measure of fiber dispersion, and ***L***_***i***_ represents fiber slack length.

To frame our model of tendon adaptation in accordance with the known abovementioned tendon physiological process, the following computational sub-models are developed: (i) a discretized fiber model of tendon mechanics, Fig 2(a), from which the force-extension behavior of the whole tendon can be estimated, Fig 2(b). (ii) a simplified model of the musculotendon (Achilles-soleus) unit, Fig 2(c), from which the metabolic cost of the (effective) soleus muscle mechanical work at the ankle joint is estimated, Fig 2(d). (iii) a model for calculating the intensity of daily load as a function of metabolic cost, Fig 2(e), (iv) models for mechanical damage and repair to individual collagen fibers, Fig 2(f), and (v) models for proteolytic damage and repair to individual collagen fibers, Fig 2(g). A single sequential pass through these sub-models, results in a remodeled tendon geometry, with a modified force-extension response, Fig 2(h). In other words, the model tendon adapts by repeatedly cycling through this algorithm. We now describe each of these sub-models in detail.

### Tendon Mechanical Model

In principle, either the collagen fibrils or the primary collagen fibers may be the anatomical units regarded as the load carrying ‘string elements’ in our model. However to render the computations more tractable, here we have chosen to represent the primary collagen fibers as the discretized string elements. For the rest of this paper, collagen fibers in our model refer to primary collagen fibers.

Human Achilles tendon cross-sectional area (CSA) at the mid-section is reported to range from under 50 mm^2^ [51, 52] to values higher than 80 mm^2^ [38, 53]. Assuming an Achilles tendon to have a CSA of 60 mm^2^ and assuming a circular cross-section for collagen fibers with an average fiber diameter of 28 μm [54], the total number of primary collagen fibers (*N*_total_) in human Achilles tendon is estimated to be:

$$N_{\text{total}}=\frac{A_{\text{T}}}{A_{\text{F}}}\approx \;100,000\;\;\text{(fibers)}$$ (1)

where *A*_T_ and *A*_F_ denote whole tendon cross-sectional area and average collagen fiber cross-sectional area In the present study, we explicitly model 100,000 collagen fibers that are of non-uniform length, but otherwise similar. We note here that it may be appropriate to have fibers with variable stiffness along their length, as it has been reported that the ‘free’ Achilles tendon is more compliant than the aponeurosis [21, 55, 56]. Nevertheless, for simplicity we adopt a uniform stiffness along the fiber length. And for simplicity we assume a Gaussian (normal) distribution for the initial profile of fiber lengths, and show that it can approximate the non-linear stress-strain curve of a tendon, Fig 1. Starting mean fiber length is chosen to be in the range 250 to 280 mm to correspond with reported total anatomic length of human Achilles tendon, including the aponeurosis [57–60].

Choosing *L*_T_ as the mean length of the tendon and *L*_*i*_ the slack length of the *i*^th^ fiber, then the linear extension of the *i*^th^ fiber (Δ*L*_*i*_) as the whole tendon undergoes linear extension Δ*L*_T_ is calculated from:

$$\Delta L_{i}=\;\{\begin{array}{cc} L_{i}-\left(L_{\text{T}}+\Delta L_{\text{T}}\right) & \text{if }L_{\text{T}}+\Delta L_{\text{T}}>L_{i}\\ 0 & \text{if }L_{\text{T}}+\Delta L_{\text{T}}\leq L_{i} \end{array}$$ (2)

For convenience, we take mean of the slack lengths of all fibers to represent the mean tendon length ($L_{\text{T}}\approx {\bar{L}}_{i}$). While the measured estimates vary, the commonly reported Young’s modulus of the Achilles tendon (*E*_T_) is about 1 GPa [61, 62]. Assuming homogenous material properties, and uniform cross-sectional area (*A*_F_) for all collagen fibers, the stiffness of the *i*^th^ fiber (*k*_*i*_) is then estimated by:

$$k_{i}=\frac{A_{\text{F}}\cdot E_{\text{T}}}{L_{i}}$$ (3)

Using Eqs (2) and (3), fiber force (*F*_*i*_) and whole tendon force (*F*_T_) at a given tendon extension can then be calculated by:

$$F_{i}=k_{i}\cdot \Delta L_{i}$$ (4)

$$F_{\text{T}}=\Sigma_{i=1}^{N_{total}}F_{i}$$ (5)

### Mechanical Damage and Repair Models

Repeated cyclic loading of tendon during daily activity damages collagen fibers [38, 63]. Our estimates for the likelihood of mechanical fatigue damage of primary collagen fibers is based on the empirical fatigue damage data for the whole human Achilles tendon obtained by Wren et al [38]. The implicit assumption we employ to use this data is that the whole Achilles tendon fatigue behavior is also representative of the individual primary collagen fibers fatigue behavior making up the human Achilles tendon. The assumption that each part of the Achilles tendon is similar to all others is likely to be a reasonable assumption for healthy tendon, but we note this is less likely to be a reasonable assumption for diseased tendon.

**Fig 3:**
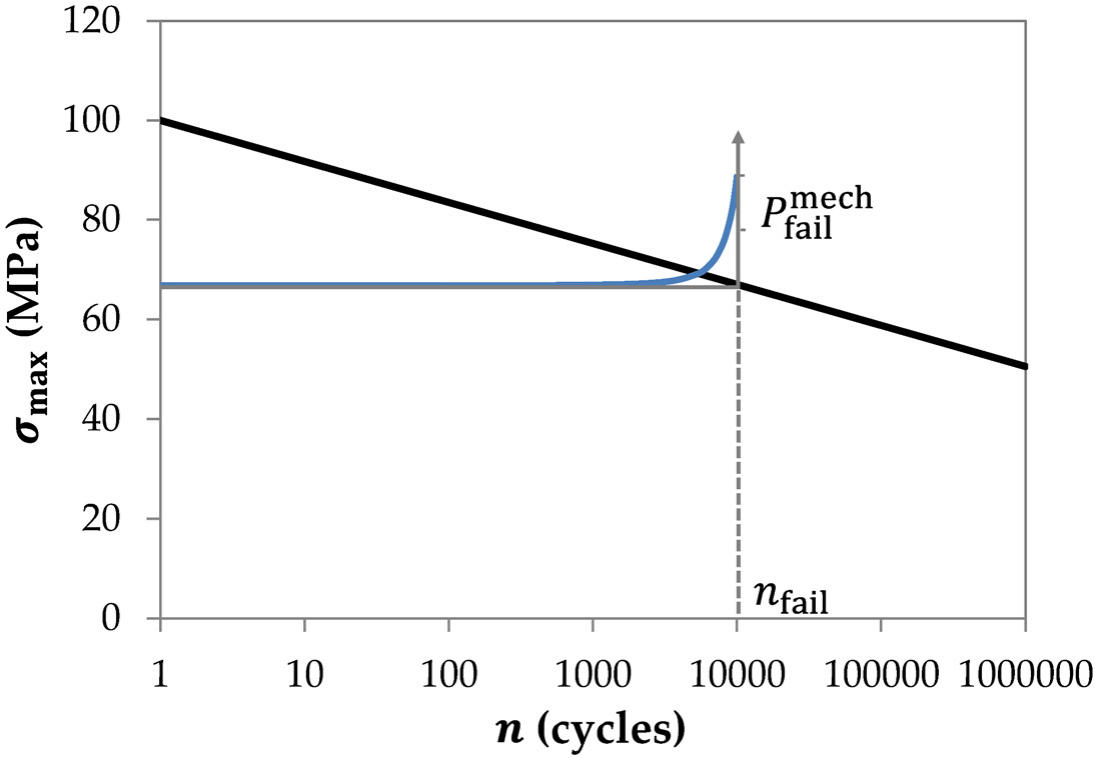
Normalized fatigue curve for whole tendon. The fatigue curve is constructed by rescaling the empirical data from [38] so the ultimate tensile strength equals the *in vivo* failure stress reported for young adults, 100 MPa [64–66]. Cumulative damage to collagen fibers is based on fitting a commonly adopted exponential failure function to typical fatigue test data on human Achilles tendon.

The average age of the 25 human subjects from whom the Achilles tendon samples were obtained by Wren et al (2003) was 75 (±12) years [38]. Therefore, in order to represent the *in vivo* damage in young adults, we chose to rescale the ultimate tensile stress value of 70 MPa reported by Wren et al (2003) [38] to 100 MPa [64–66], while leaving the slope of the fatigue curve unchanged. Fig 3 shows the normalized fatigue curve for collagen fibers employed in our model. This rescaled human Achilles tendon fatigue curve is probably more representative of younger adults, though other scalings may be deemed appropriate depending on data and the intended purpose of the model. However provided that reasonable values are chosen, the actual values for scaling are not critical, and do not substantially change the findings reported here (see later sensitivity analysis).

From Fig 3, the number of loading cycles to tendon failure (*n*_fail_) at a given peak fiber stress (*σ*_max_) are calculated using:

$$\sigma_{\text{max}}=a-b\cdot {\text{log}}_{10}\left(n_{\text{fail}}\right)$$ (6)

$$∴n_{\text{fail}}=10^{\frac{a-\sigma_{\text{max}}}{b}}$$ (7)

where *a* corresponds to the ultimate tensile stress at one cycle, in this case 100 (MPa), and *b* is the slope of the logarithmic fatigue curve in Fig 3, in this case 8.25 (MPa/log(*n*)). Peak tensile stress of the *i*^th^ fiber ($\sigma_{\text{max}}^{i}$) in a tendon undergo)ing linear extension Δ*L*_T_ is calculated by:

$$\sigma_{\text{max}}^{i}=E_{\text{T}}\cdot \frac{\Delta L_{i}}{L_{i}}$$ (8)

where fiber extension Δ*L*_*i*_ is calculated from Eq 2. It is clear that typical daily activities lead to peak stress levels that rarely (if ever) result in complete failure of a normal tendon. Consequently we need to devise a ‘cumulative damage function’ to estimate the amount of damage arising from daily activity. For our ‘string’ tendon model, cumulative tendon damage is assumed to be directly proportional to the fraction of broken fibers. The fraction of broken fibers as a result of daily activity can be estimated from a failure (or reliability) function for individual collagen fibers. However due to the lack of experimental data on failure functions ($P_{fail}^{\text{mech}}$) or reliability functions ($R=1-P_{fail}^{\text{mech}}$) for tendon, we employ a commonly adopted ‘exponential failure function’ [67] to describe focal damage failure of individual collagen fibers within the Achilles tendon. Therefore the probability of mechanical failure of an individual fiber experiencing peak fiber stress *σ*_max_ and *n* load cycles is estimated by:

$$P_{fail}^{\text{mech}}=-\kappa +\kappa \cdot e^{\lambda \;\frac{n}{n_{\text{fail}}}}$$ (9)

The fitting constants *κ* and *λ* in (Eq 9) are defined such that $P_{fail}^{\text{mech}}=0$ at = 0, $P_{fail}^{\text{mech}}=0.1$ at *n* = *n*_fail_/2 and $P_{fail}^{\text{mech}}=1$ at *n* = *n*_fail_. These fitting constants are chosen based on reported typical cyclic fatigue test on human Achilles tendon reported in Fig 2(b) and 2(c) of Wren et al (2003) [38]. Fitting Eq 9 to this figure suggests reasonable parameter values are, *κ* = 0.0125 and *λ* = 4.395. A typical cumulative damage probability curve is shown in Fig 3. However clearly these fitting constants can be adjusted to fit experimental results as required, while the influence of these parameters on our model outputs are quantitated in a later sensitivity analysis.

**Fig 4:**
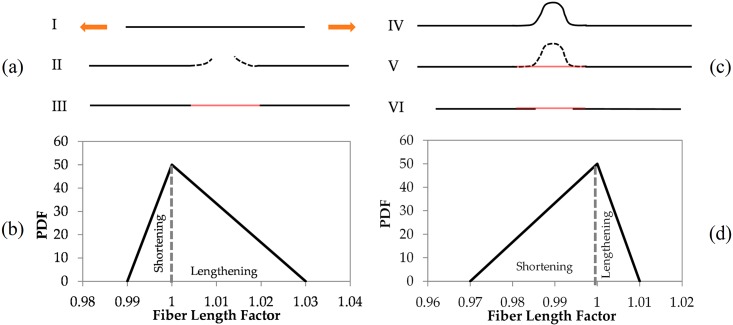
Collagen fiber remodeling. a) remodeling of collagen fibers by mechanical damage and repair, (I) shorter fibers are subject to higher strains, (II) fiber focal damage under mechanical strain forming a gap, (III) fiber repair at a longer length by filling in the gap with new collagen, b) repair probability density function following mechanical damage, quantifies the bias toward fiber lengthening, c) remodeling of collagen fiber by proteolytic damage and repair, (IV) longer fibers are subject to lower strains, thus more likely to be degraded by proteases, (V) new collagen forms across the gap while excess fiber is degraded resulting in a shorter fiber (VI), c) repair probability density function following proteolytic damage quantifies the bias towards fiber shortening.

In our model for an Achilles tendon with normal physiology, if a fiber mechanically fails, it is always repaired (which may not happen in a diseased tendon). A repaired fiber may (probabilistically) be repaired either shorter or longer, however, we bias the repair of mechanically damaged fibers towards lengthening (Fig 4). A probabilistic interpretation of fiber repair as used in our model is depicted in Fig 4(b), which shows the probability distribution of relative length changes to a fiber following its repair.

We suggest that this repaired length change, depicted Fig 4(a), is consistent with the following conceptual model of the repair process following mechanical damage. First, the two ends of the broken fiber are enzymatically debrided by proteases to obtain a suitable undamaged surface from which a new portion of collagen fiber can be constructed. A new portion of collagen fiber is then created by polymerization of tropocollagen molecules [68, 69]. While the section of fiber debrided may be longer than the newly formed portion of collagen fiber, leading to further fiber shortening, on average the broken ends are more likely to lie somewhat apart, so the new portion of fiber bridges both this gap and any fiber debridement, and so the repaired mechanically damaged fiber is on average longer. The gap between the broken ends arises at least partly because mechanically damaged fibers are on average shorter than remaining nearby fibers, but it seems plausible that the gap between broken fiber ends may also be partly promoted by other events, such as the elastic recoil of the fractured ends of a failed fiber or subsequent cyclic friction forces between fibers. The gap between the broken ends is observable in SEM images of damaged collagenous matrices [43, 70, 71]. A schematic depiction of this is shown in Fig 4(a). It has been noted previously by Provenzano et al [43], that if the gaps between the fractured ends are filled by newly polymerized collagen fiber, then the repaired fibers lengths are increased.

### Proteolytic Damage and Repair

Proteases remove damaged or unwanted ECM as part of normal tissue turnover and collagen fiber homeostasis [14, 23, 41], but the rate of collagen degradation is modified by collagen strain. For example, Wyatt el al. [72] reports an almost complete cessation in collagenase degradation rate when rat tail fascicles were strained 4–5% [72]. However, Flynn et al. performed similar tests on single collagen fibrils, thereby avoiding rotational deformations of collagen fibers during extension (rotational deformations are often observed in collagen networks made up of fibrils with a variety of fibril orientations) [73]. For the experimental tests reported by Flynn et al (2013), which probably most closely approximate the (linear) fibril structure observed in Achilles tendon, collagenase degradation of fibrils is prevented at strains larger than about 1.5%.

**Fig 5:**
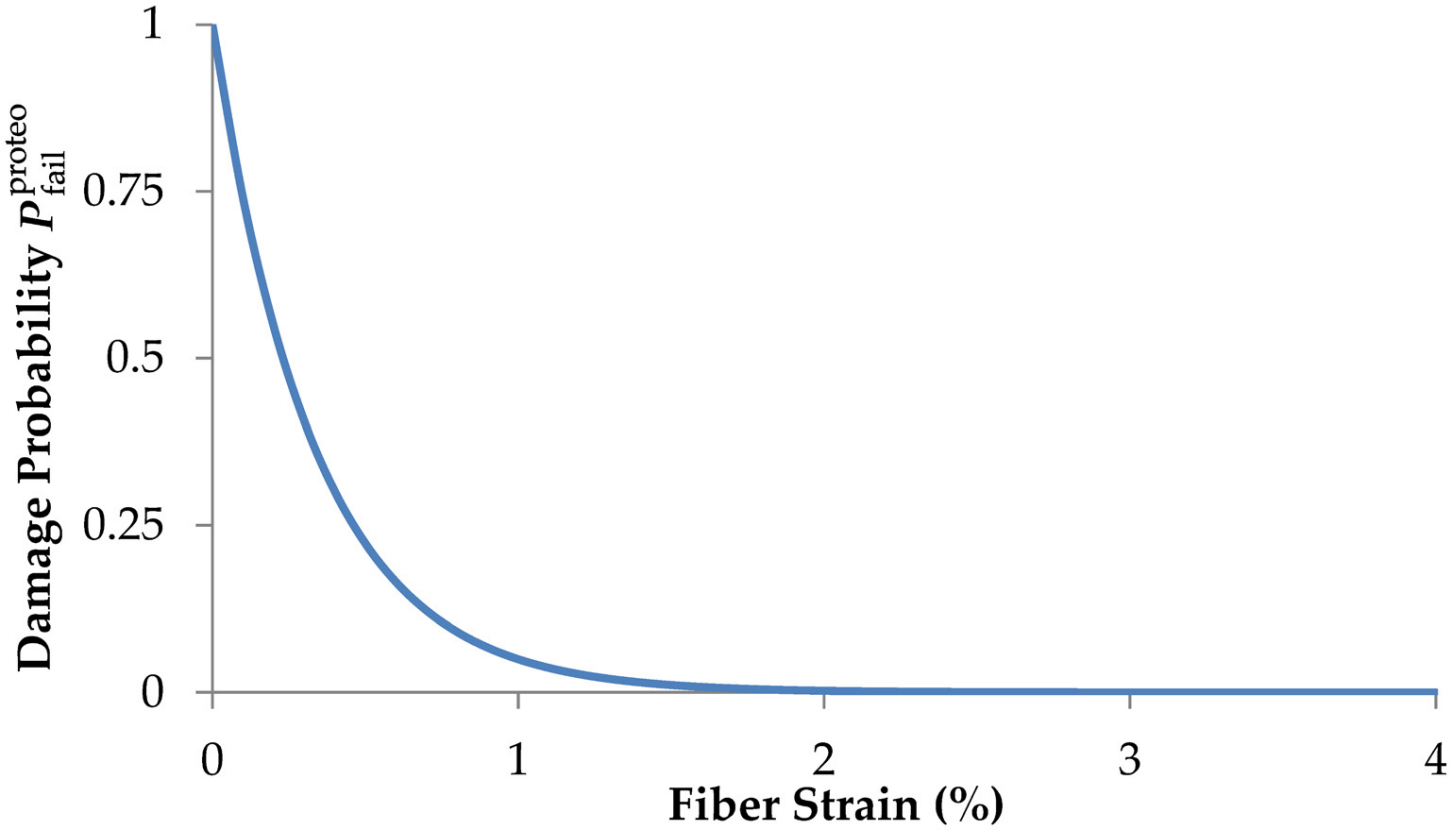
Probability function of fiber proteolytic damage as function of fiber strain. Fibers are completely shielded from proteolytic damage for peak fiber strains *ε*_max_ ≥ 1.5%.

These test results suggest that in terms of our ‘string’ model of tendon, for a given tendon strain, relatively long collagen fibers are less stretched along their length, which render them more susceptible to being degraded by active proteases [22, 41, 48, 74–76]. Based on the experimental results of Flynn et al [47], we employ an exponentially decreasing probability of fiber cleavage with increasing strain (Fig 5), viz:

$$P_{fail}^{\text{proteo}}=e^{-\phi \cdot \varepsilon_{\text{max}}}$$ (10)

where *ε*_max_ is the fiber peak strain during a gait cycle and *ϕ* is a fitting constant. To accord with the observations of Flynn et al. [73], *ϕ* is calculated to be 300. This selection of *ϕ* results in almost no proteolytic damage in fibers experiencing peak strains *ε*_max_ ≥ 1.5%. Clearly the sensitivity to proteolytic damage can be altered by varying the constant.

It is equally clear that Eq 10 is a very crude modeling representation of an actual proteolytic process occurring within tendon, as binding of MMPs to collagen fibers, the movement of MMPs along collagen fibers, the surface state of the collagen fibers, and the history of cyclic strain experienced by the collagen fibers, are all time dependent, and so the level of protection afforded to collagen fibers must also be time dependent. A more sophisticated proteolytic damage model would include such time dependencies, but unfortunately to date there is little data available in the literature to suggest more precise functional relationships.

When proteolytic degradation of the collagen fiber is complete, once again new collagen molecules polymerize to create newly formed collagen fiber, which bridges the gap between the degraded ends (see schematic Fig 4(c)). For each fiber that is proteolytically degraded, the fiber repair model determines a probabilistic repair length. As in the case of mechanical damage, the repair length is found by sampling a triangular probability distribution. However, this time the triangular probability distribution has a greater tendency to shorten the original fiber, see Fig 4(d). In other words, the fiber section removed proteolytically is on average longer than that filled by newly formed fiber section, and so the repaired fiber is shortened. We note that the mechanism for fiber shortening is not known with certainty. However, it is possible that passive mechanical forces may contribute to fiber shortening (e.g. cyclic frictional forces, or compressive residual stresses in long fibers may relax), or active forces generated by cells may contribute to fiber shortening (e.g. a number of experiments have reported that tenocyte generated contractile forces can actively shorten collagen fibers [41, 74, 75, 77].

### Integration of the Tendon Model with the Muscle Model

To provide an appropriate context to test adaptation of Achilles tendon to its loading environment, we set our new tendon model within a standard three-component Hill-type model. Representing the musculotendon unit, the Hill-type model is composed of a contractile element and two elastic elements, Fig 2(c). The contractile element and the parallel elastic element simulate the integrative behavior of the human soleus through gait cycles [78, 79]. The soleus is the muscle of choice for examining our tendon model for the following reasons: (i) among the plantarflexor muscles it is the largest muscle, and from several modeling studies it has become clear that the soleus is the primary muscle responsible for producing ankle power and work in both walking and running [80–82]. Furthermore, modeling results has found the soleus to be among the most important producers of mechanical work during walking and running across all lower limb muscles [83, 84]. (ii) The soleus only crosses the ankle, unlike the gastrocnemius muscles that cross both the ankle and knee joints (knee flexor). This simplifies the modeling of muscle force using our hill-type model, eliminating potentially complicating factors.

Joint torque sharing between the soleus and the other synergist muscles is simplified by initially attributing the torque produced by the soleus to the relative physiological cross sectional area of the soleus and the other ankle plantarflexors combined. Soleus force is subsequently computed from a joint angle-specific soleus moment arm [59]. The activation required to produce this force is modelled incorporating muscle force-length-velocity constraints. Muscle fiber lengths and velocities are influenced in our calculation by ankle joint angle, muscle pennation angle (we assumed a constant volume muscle model) as well as tendon stretch.

The series elastic element in the musculotendon unit shown in Fig 2(c) represents the Achilles tendon, with its mechanical properties obtained from the discretized tendon model described above. As we allow tendon remodeling over time, mechanical properties of this series elastic element, representing the Achilles tendon, also changes over time.

For simplicity the contractile element and its parallel elastic element are taken to have constant properties in all our simulations, though in reality these may also adapt over time [85, 86]. This simplification is to focus our attention on the adaptation process of the Achilles tendon alone, and to exclude muscle adaptation (which has been investigated elsewhere [85, 86]). Allowing the muscle to adapt simultaneously with the tendon could potentially complicate our understanding of tendon adaptation, and possibly obscure tendon responses that are of interest here. But clearly muscle does adapt too, and inclusion of such mechanisms is an obvious extension for developing a more realistic future model.

**Fig 6:**
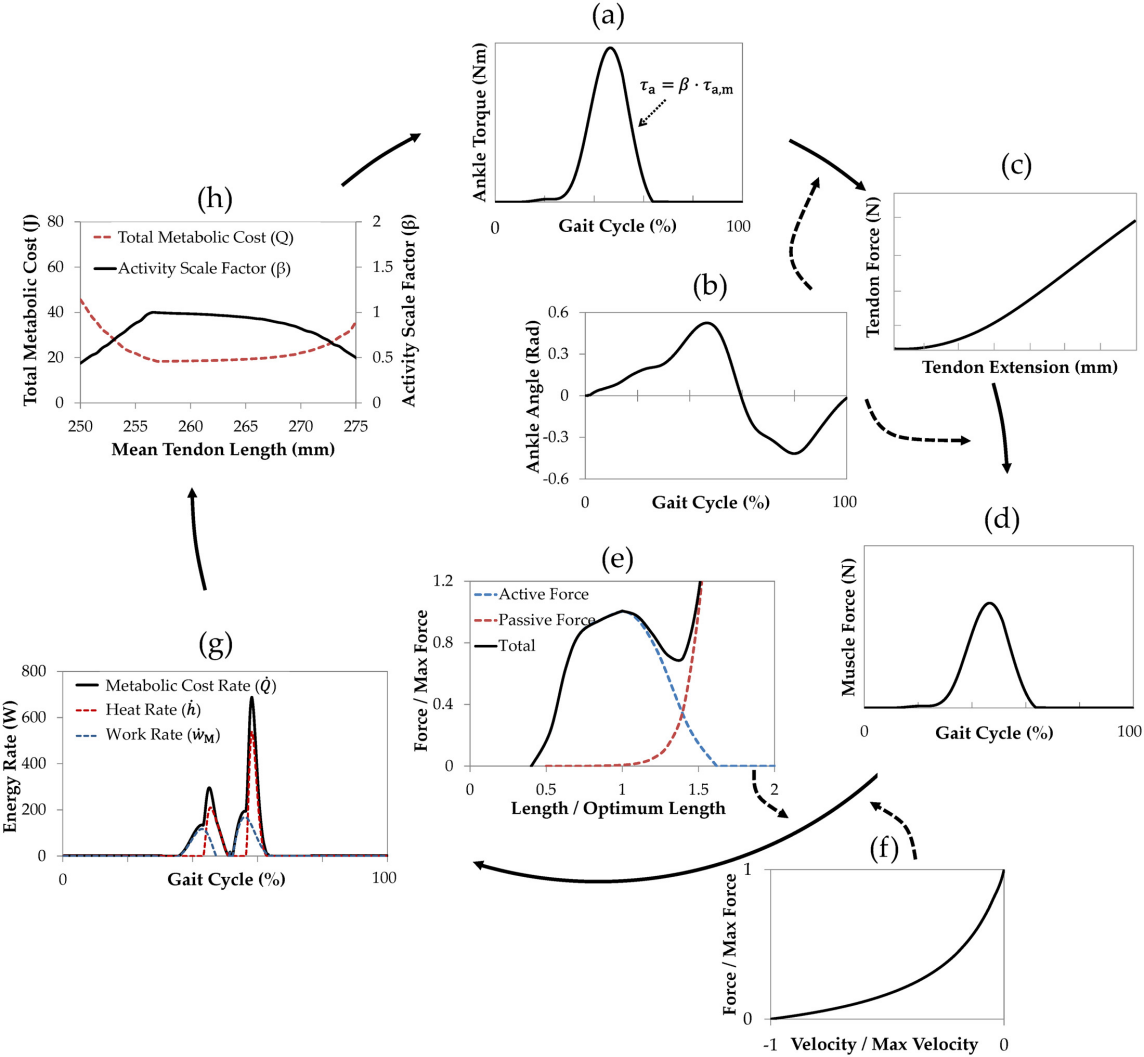
Musculotendon operation. a) ankle torque over a gait cycle, b) ankle angle over a gait cycle, c) force-extension response of tendon from our discrete tendon model, d) required muscle force to produce the ankle torques in part (a), e) muscle length-force relation, f) muscle force-velocity relation, g) metabolic cost rate as a combination of generated muscle heat and mechanical work, h) ankle torque adjustment according to the ratio of the current total metabolic cost to the minimum total metabolic cost for a range of mean tendon lengths and fiber length standard deviations (SDs). Total metabolic cost results in part (h) are plotted for mean tendon lengths between 250mm to 275mm and fiber length SD = 1.5 mm.

Fig 6 illustrates the algorithm for updating the tendon properties in the musculotendon model as a result of activity. The musculotendon model uses an inverse dynamics approach with ankle torque and kinematics as inputs to calculate the required muscle force. The ankle torque and kinematics were experimentally obtained from motion tracking and force measurements from an adult subject during walking [87].

The algorithmic steps in Fig 6 include: ankle torque, Fig 6(a), and musculoskeletal dimensions (moment arms) and ankle angles, Fig 6(b) are employed to first calculate tendon force. The tendon model response to force is then used to calculate tendon extension, Fig 6(c). Tendon extension and ankle angles, Fig 6(b) and 6(c), are then used to calculate muscle fiber pennation angle, muscle fiber operating length and muscle force (*F*_M_), Fig 6(d), which balances the calculated tendon force. Muscle activation (*M*_act_) to generate the required muscle force is estimated based on muscle length, Fig 6(e) [79], and muscle velocity, Fig 6(f) [79, 88]. The expression used to calculate muscle activation, based on formulations by Buchanan et al. [79], is:

$$M_{\text{act}}=\frac{F_{\text{M}}}{F_{\text{L}}\cdot F_{\text{V}}\cdot F_{\text{M,max}}}$$ (11)

*M*_act_ (muscle activation) is a dimensionless number between zero and one that represents the fraction of maximal muscle activation. *F*_M_ is the muscle force, Fig 6(d), while *F*_L_ is the muscle force at current length as a fraction of the maximum isometric force (*F*_M,max_), Fig 6(e), and *F*_V_ is the muscle force at the current contraction velocity as a fraction of the maximum isometric force (*F*_M,max_), Fig 6(f). The muscle activation *M*_act_ from Eq (10) is then used to calculate the metabolic cost rate (power), and integration of this quantity with respect to time gives the total metabolic cost during a gait cycle, as described in [78].

The metabolic cost rate ($\dot{Q}$) during a gait cycle, Fig 6(g), is expressed as the sum of four terms: activation heat rate (${\dot{h}}_{\text{act}}$), maintenance heat rate (${\dot{h}}_{\text{m}}$), shortening/lengthening heat rate (${\dot{h}}_{\text{sl}}$) and the mechanical work rate performed by the muscle (${\dot{w}}_{\text{M}}$) [78], viz:

$$\dot{Q}={\dot{h}}_{\text{act}}+{\dot{h}}_{\text{m}}+{\dot{h}}_{\text{sl}}+{\dot{w}}_{\text{M}}$$ (12)

Now that we have defined our musculotendon unit model, we need to recognize that the musculotendon unit does not operate in isolation from the whole organism, but is in fact part of the whole organism. This relationship between the musculotendon unit and whole organism puts constraints on the muscultotendon units operation, which help guide the tendon to an *in vivo* equilibrium state. Potential factors at the whole organism level affecting the musculotendon operation in the adult include sensory feedback signaling (including pain), neural muscle activation patterning, higher order cognitive inputs (e.g. willpower) and oxygen and metabolic energy availability from the organism to make sure there is matching of supply-demand functions over the whole musculotendon unit.

The equilibrium state achieved by tendon operating within the musculotendon unit, operating within the whole organism, depends on these interactions/constraints, which may be formulated mathematically as optimization of a multi-objective function. Clearly this multi-objective function can vary over time with changes in environmental and sensory inputs, nutritional status and determination of the individual, and it is these changes that usually drive tendon adaptation *in vivo*. But for our modeling purposes, how can we simply and reasonably take into account this substantial *in vivo* complexity?

At equilibrium it is likely that for everyday repetitive activities such as walking, an important contributor to the multi-objective function is musculotendon unit economy. For habitual repetitive activities such as walking, energy minimization is regarded by some as a key optimization criterion dictating locomotor behavior [89, 90]. There is much evidence pointing to movement patterns that minimize energy expenditure, from the selection of preferred walking speeds in humans and other species [91, 92] to preferred stride frequencies [93, 94] and preferred gaits [95, 96].

Consequently, it is likely that Achilles-soleus unit economy is close to being maximized when the tendon geometry has reached its geometrical equilibrium state, and that Achilles-soleus unit economy will ‘fall’ on either side of the tendon’s geometrical equilibrium state. Making these assumptions, the simplest way for us to approximate the change in walking economy with tendon geometry in our model is to reflect the effect of current metabolic cost on the musculotendon unit load intensity. This can be implemented most simply by scaling the ankle torque while keeping both gait pattern and number of load cycles per day constant. Taking this approach effectively acts as a constraint on the musculotendon unit operation, preventing the total metabolic cost from becoming physiologically unrealistic at some tendon geometries.

Therefore ankle torque (*τ*_a_) can be calculated via:

$$\tau_{\text{a}}=\beta \cdot \tau_{\text{a},\text{m}}$$ (13)

where *τ*_*a*,*m*_ is the lab-measured ankle torque and *β* is an activity scale factor calculated by:

$$\beta =\frac{Q_{\text{min}}}{Q}$$ (14)

where *Q* refers to the total metabolic cost of muscle activation and *Q*_min_ refers to the minimum total metabolic cost of muscle activation. Fig 6(h), shows the relationship between the activity scale factor (0 < *β* ≤ 1) and the metabolic cost for a range of tendon lengths and constant fiber length dispersion. The calculated activity scale factor *β* is used in the next cycle to determine subsequent ankle torques, from which flows tendon forces, muscle forces and metabolic cost, which are calculated via cycling through the algorithm for muscultotendon unit operation depicted in Fig 6. Clearly a more realistic model would take into account changes in the multi-objective function governing musculotendon unit interactions/constraints with the whole body in a much more sophisticated way, and also involve changes in both gait patterns and number of load cycles. Relaxing the assumptions made here represent an interesting direction for future research.

We chose a single cycle of remodeling, shown in Fig 2, to represent a 24-hour period. To correspond the tendon activity level with this time-frame, we subjected all our tendon models to a total of n = 5,000 load cycles per day, which approximates the number of gait cycles of active adults [97–99].

To reduce possible time discretization errors, the tendon model is subjected to loading cycles in three equally spaced blocks of simulated activity cycles during a day. At the end of an activity block, the tendon’s fatigue damage is assessed as outlined above, resulting in loss of some intact fibers. With an updated metabolic cost, tendon peak force for the next loading block is updated wherein tendon continues undergoing mechanical loading. At the end of the third loading block, proteolytic damage and finally repair of the mechanically and proteolytically damaged fibers take place, as previously explained, resulting in a new fiber length distribution (and restored fiber number). This daily cycle is repeated to simulate tendon remodeling over weeks or months, and adaptation of tendon properties can be tracked over time.

A summary of parameter symbols and values used in the model are shown in Table 1 below.

**Table 1:** Parameter names, symbols and values.

Parameter Name	Parameter Symbol	Parameter Value
Number of fibers	*N*_*total*_	100,000
Average collagen fiber CSA	*A*_*F*_	6.15 × 10^−4^ mm^2^
Average tendon CSA	*A*_*T*_	60mm^2^
Young’s modulus of tendon	*E*_*T*_	1GPa
Loading cycles per day	*n*	5,000
Tendon ultimate tensile stress	*a*	100MPa
Logarithmic fatigue curve slope	*b*	8.25MPa/log(*n*_fail_)
Peak ankle torque, measured	*τ*_a,m_	165Nm

## Results

**Fig 7:**
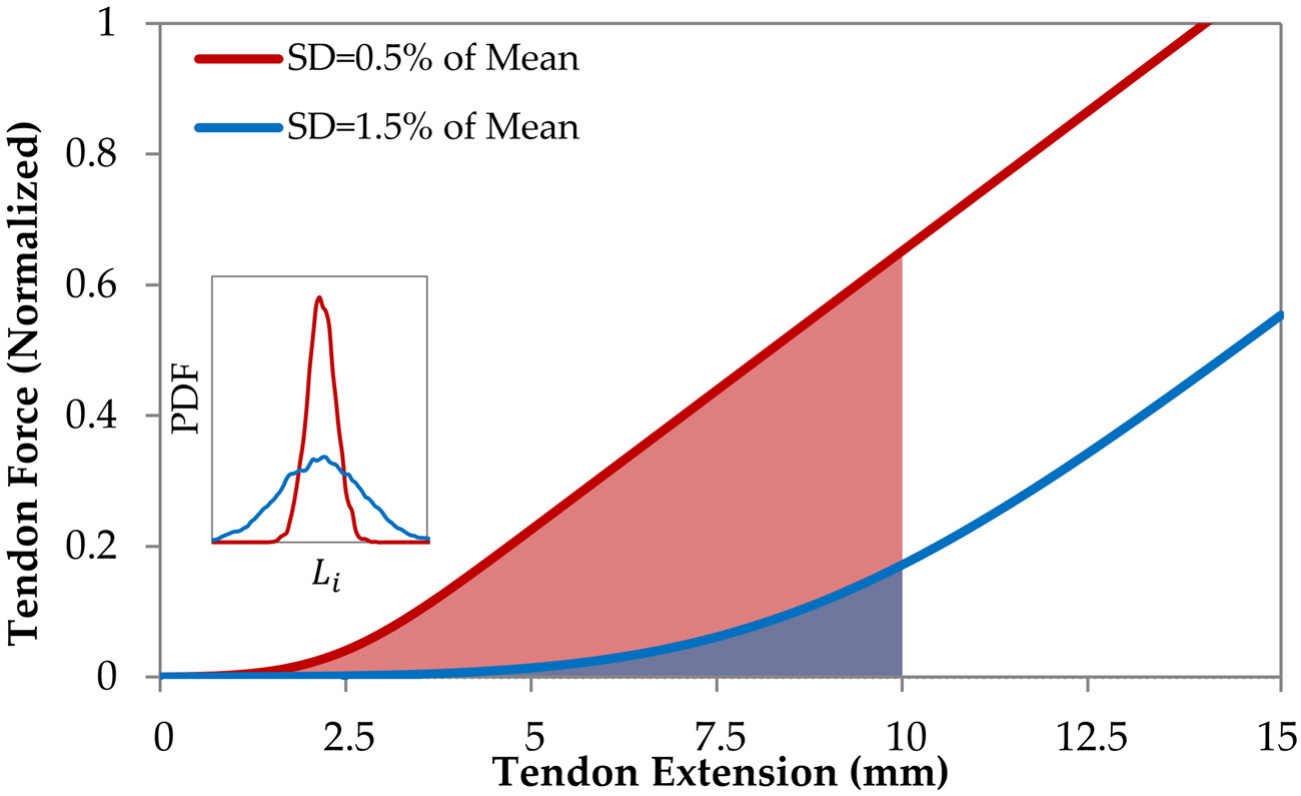
Mechanical response of tendons with different fiber length distributions. Force-extension curves for tendons with identical mean fiber length and different length standard deviations (SDs) i.e. SDs = 0.5% and 1.5% of mean fiber length.

We first consider fiber length distribution of the model tendon, as fiber dispersion strongly influences its mechanical properties. A model tendon with reduced fiber length dispersion (i.e. reduced standard deviation) experiences more rapid fiber recruitment and force development with strain, while a tendon with a larger fiber length dispersion (i.e. larger standard deviation) exhibits slower fiber recruitment and force development with strain (see Fig 7). Strain energy is the potential energy stored by elastic materials as they undergo deformation. For elastic materials such as tendon, it is quantified as the dot product of force and displacement. The elastic strain energy is equal to the area under the force-extension curves shown in Fig 7. For a given tendon extension of two otherwise identical tendons, the tendon with smaller fiber dispersion has a higher force and more strain energy is stored. However for constant force, the tendon with the lower stiffness will store more strain energy [7].

**Fig 8:**
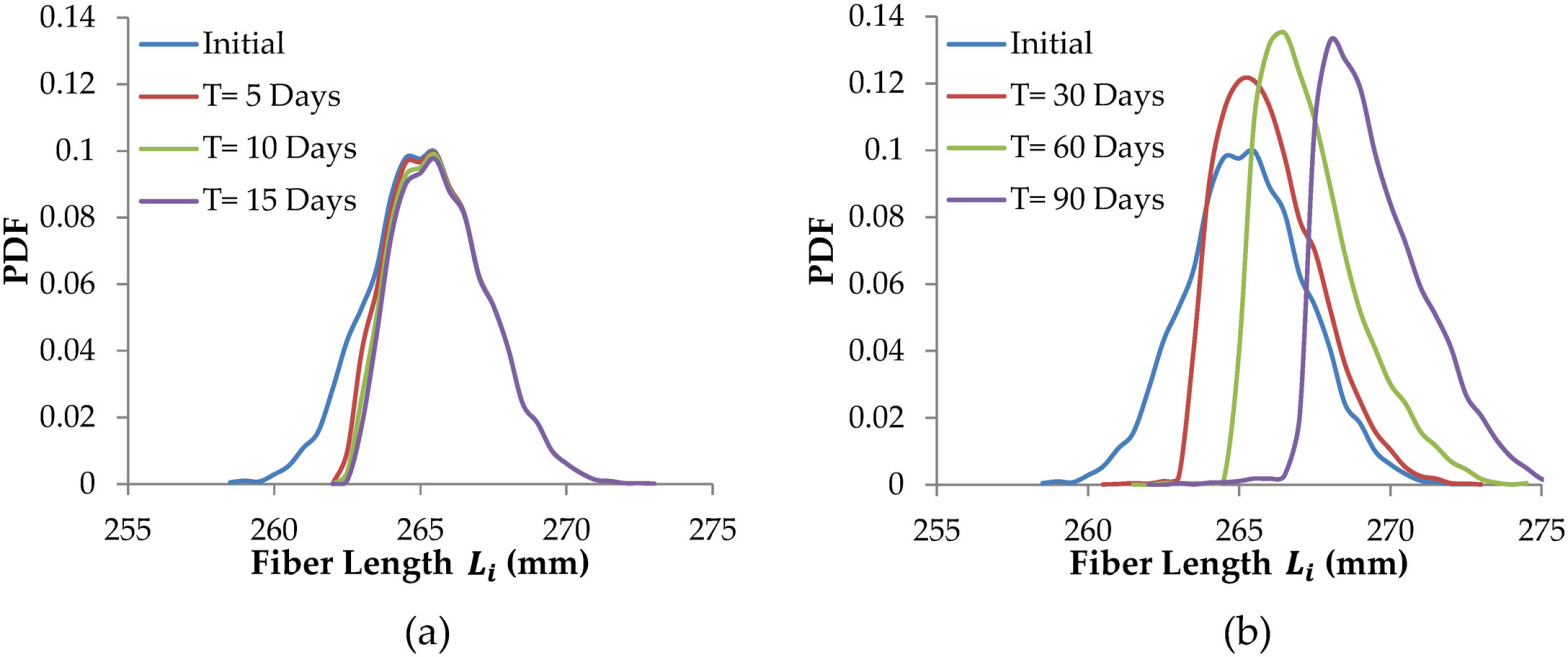
Tendon remodeling by mechanical damage and repair. a) tendon remodeling by mechanical damage alone over 15 days, b) tendon remodeling by mechanical damage and repair over 90 days. Tendon peak force and number of loading cycles are kept constant for all simulated days.

**Fig 9:**
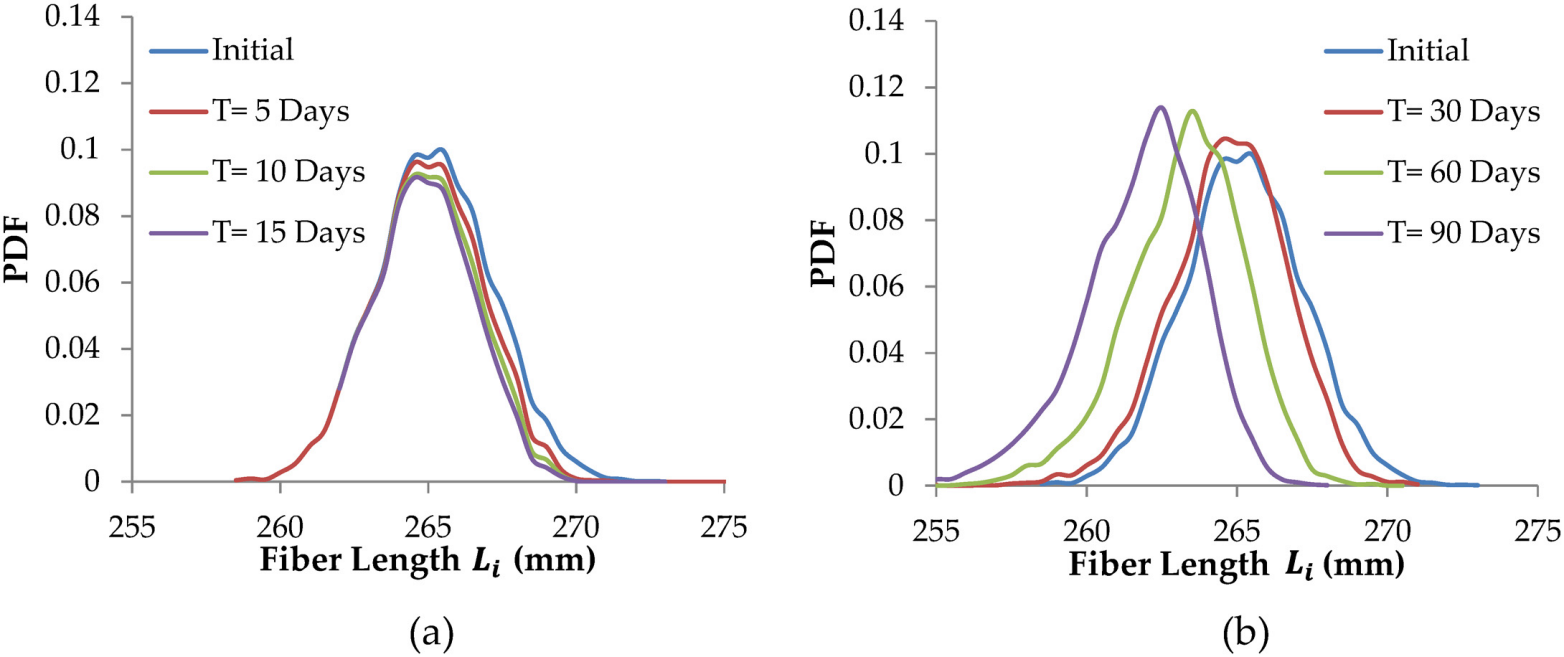
Tendon remodeling by proteolytic damage and repair. (a) tendon remodeling by proteolytic damage alone over 15 days, b) tendon remodeling by proteolytic damage and repair over 90 days. Tendon peak force and number of loading cycles are kept constant for all simulated days.

The effects of damage and repair models on tendon fiber length distributions are next demonstrated in Figs 8 and 9. All tendon simulation results shown in these two figures are for cyclic loading n = 5,000 cycles/day and a peak stress of 55 MPa (i.e. 5.5% strain). The model tendons initially have a normal distribution of fiber lengths, with mean fiber length 275mm and fiber length standard deviation of 2mm.

Fig 8(a) represents remodeling of tendon by mechanical damage only (i.e. there is no repair operating). The shortest fibers, where fiber strain is the highest, are damaged rapidly and so break first (they are not repaired, as no repair is operating). The remaining shortest fiber are now more abundant (as the probability density function of fiber lengths progressively increases up to the mean), which slows the rate of advance of the broken fiber. Note that without including subsequent repair, the total number of intact fibers gradually decreases over time. If mechanical fatigue damage continues for a long time without repair, eventually all the fibers would fail, and the tendon would rupture [38].

In Fig 8(b) both mechanical damage and repair are operating, so the total number of fibers remains constant. However, repaired lengths are on average longer than the original fiber slack lengths, so the mean fiber length increases, and the whole fiber population ‘marches’ towards longer tendon lengths (see Fig 8(b)). We note that standard deviation of fiber lengths initially changes, but becomes relatively constant over time, even as the population of fibers marches towards longer tendon lengths.

Fig 9(a) represents remodeling of a tendon by only proteolytic damage (i.e. there is no repair operating). The longest fibers, where strain is the low, undergo rapid proteolytic damage. The remaining shorter fibers of the population experience progressively higher strains, which helps preserve them. As fibers are removed proteolytically there are fewer fibers and so they experience higher the average strain, which slows their removal.

In Fig 9(b), both proteolytic damage and repair processes operate. The longest fibers are first proteolytically damaged, but when repaired, on average they are shorter. Progressive damage of the longer fibers in the population combined with repair with fiber shortening results in the entire fiber distribution ‘marching’ to the left.

Results shown in Figs 8 and 9 are obtained using only the tendon model. Hereafter, activity levels are calculated by incorporating the tendon model within the Hill-type musculotendon model, as described above.

**Fig 10:**
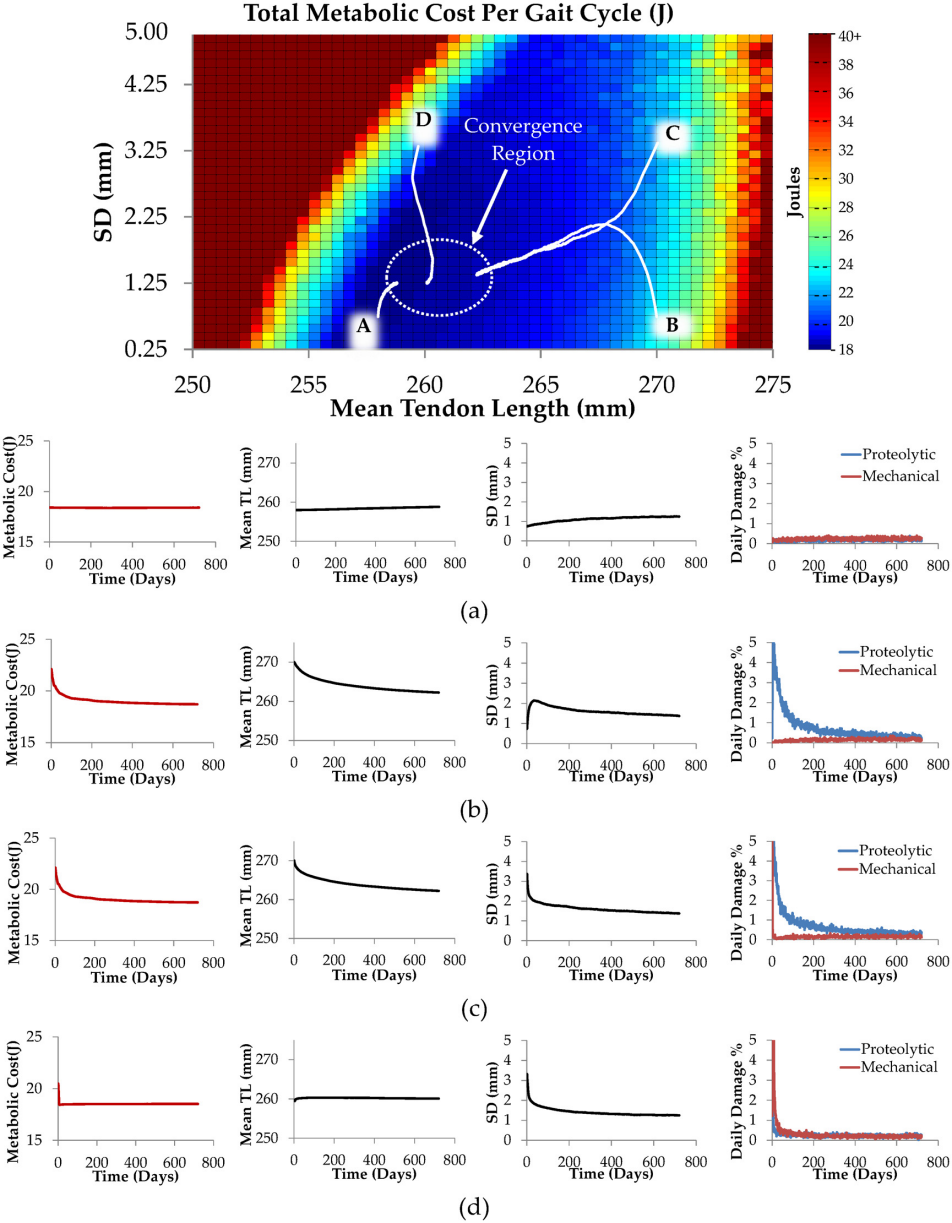
Tendon remodeling in response to metabolic cost. Tracking geometric changes in four sample tendons with initial geometries corresponding with points A, B, C and D over 720 days of simulation. a-d) Total metabolic cost, mean tendon length, fiber length standard deviation, mechanical damage rate and proteolytic damage rate plots over 720 days for paths A-D respectively.

The calculated total metabolic cost for a single gait cycle for a range of tendon slack lengths and fiber length standard deviations are shown in the color map plot of Fig 10. To illustrate tendon remodeling behavior with the musculotendon unit, we chose four arbitrary initial tendon geometries (i.e. each tendon is given a different initial tendon length and fiber dispersion). All tendons are then allowed to remodel for a period of 720 days, subjected to 5,000 loading cycles every day, and all other model parameters are held constant. Fig 10 shows remodeling paths for each of the four tendons (see paths A-D in Fig 10). Because the tendons are initially in disequilibrium states for the musculotendon unit conditions, they remodel towards their normal tendon length, which is an equilibrium state.

Importantly, we note that with appropriate selection of damage and repair parameters (as detailed in the model development described above), for each of the initially different tendon geometries illustrated in Fig 10, their remodeling paths all converge towards final equilibrium states within a region of tendon geometries. Within the assumptions of the computational model as described above, this clearly illustrates that the chosen parameters in the proposed remodeling processes are capable of directing tendon adaptation so that each tendon approaches an equilibrium geometrical state, and that this state can coincide with a region of minimum metabolic cost per gait cycle.

**Fig 11:**
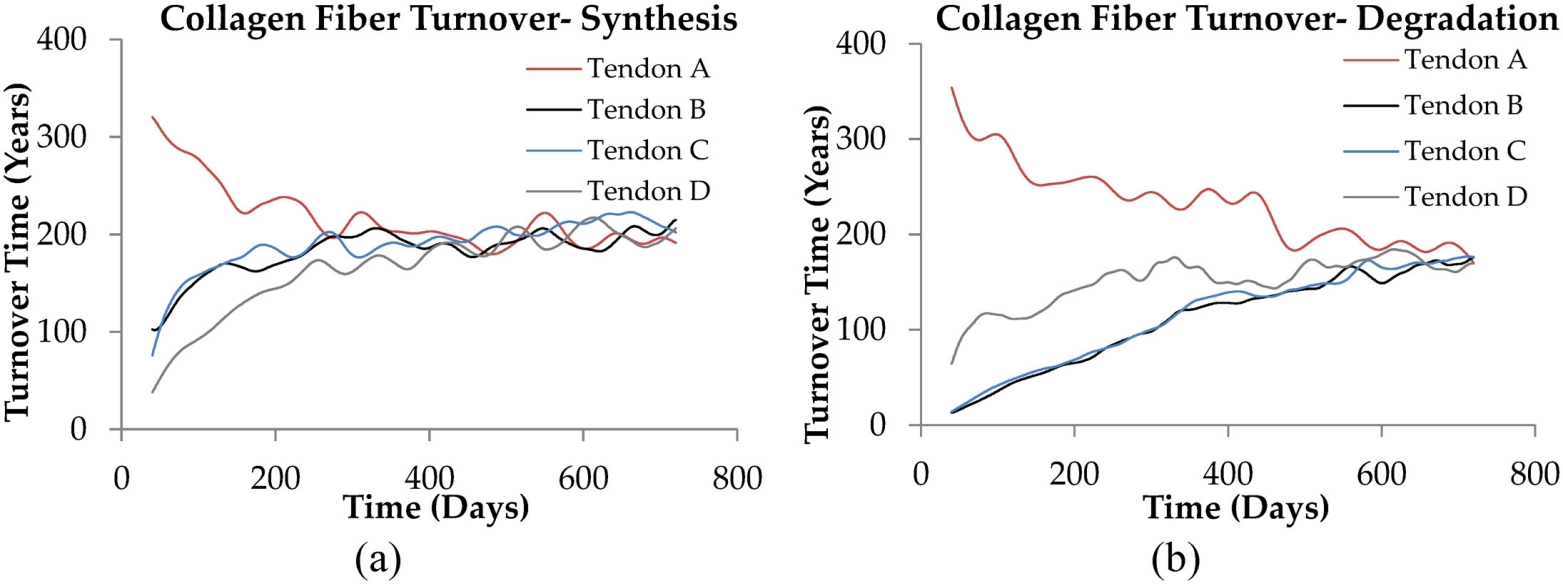
Collagen fiber turnover time during tendon remodeling. (a) Collagen fiber synthesis turnover time and (b) degradation turnover time for selected tendons of (Fig 10A–10D) during the remodeling process. Collagen fiber turnover time at equilibrium reaches around 180 years.

During the remodeling from initial states A to D shown in Fig 10, collagen fibrils are mechanically and proteolytically degraded and repaired as they remodel over time. Fig 11 shows collagen fiber degradation and synthesis turnover times during the remodeling as each tendon moves along its remodeling path (i.e. along each of the paths A to D shown in Fig 10). Collagen fiber degradation turnover time refers to the ratio of total initial fibrillar collagen content to the rate of collagen fiber removal, as a result of both mechanical and proteolytic damage processes. Collagen fiber synthesis turnover time refers to the ratio of total initial fibrillar collagen content to the rate of new fibrillar collagen formation, as a result of repair processes. At equilibrium, when there is no apparent change in tendon length or fiber dispersion, the degradation and synthesis turnover times are equal.

**Fig 12:**
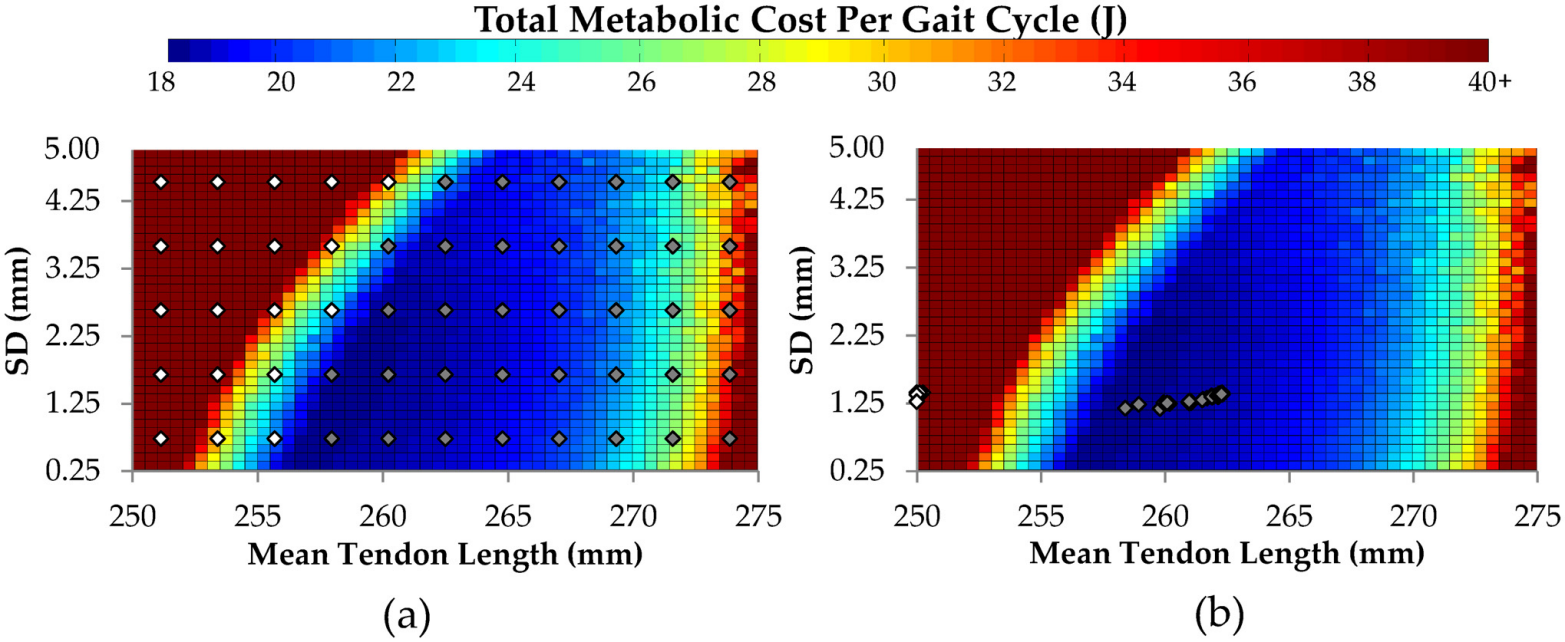
Tendon remodeling exploration. a) 55 equally spaced initial tendon geometries selected across the metabolic cost region, b) final geometries after 720 days of remodeling. White points indicate unstable initial geometries, dark points indicate initial geometries converging to an equilibrium state.

Fig 12 explores the tendon geometries after 720 days of remodeling for each of 55 equally spaced initial tendon geometries placed over the whole tendon geometry domain of tendon length and fiber dispersion while all other model parameters are held constant. Based on the tendon geometries after 720 days remodeling, these 55 initial tendon geometries can be categorized into two distinct groups. We observe that some initial geometries remodel to an equilibrium state (dark shaded points), coinciding with minimum metabolic cost, while the remaining initial geometries remodel towards non-physiological states (light shaded points), where tendons are short (and growing shorter) and metabolic costs are very high (and growing higher).

Finally, we report the sensitivity of equilibrium tendon length and dispersion to variations in model parameters. For our purpose, we define normalized sensitivity to be:

$$S_{YP}=\;\frac{1/Y_{e}\partial Y}{1/P_{e}\partial P}$$ (15)

where Y is the length of the tendon (or tendon fiber dispersion), and *P* is a model parameter. Model sensitivities are estimated by incrementing model parameters by 5%. Normalized sensitivities for fourteen model parameters are shown in Table 2.

**Table 2:** Sensitivity to changes of model parameters (% change Y/% change P, where Y is tendon length or dispersion, and P are model parameters).

Parameter (P)	Length Sensitivity	Dispersion Sensitivity
Activity Scale Factor β	0.070	-0.41
Ultimate Tensile Stress *a*	-0.069	2.1
Fatigue Slope *b*	0.031	-0.37
Mechanical Damage κ	0.0041	-0.032
Mechanical Damage λ	0.0028	-0.030
Total Daily Cycles n	0.0017	-0.0079
Proteolytic Damage φ	0.037	0.033
Repair (% Change of Length) Mean	-0.0026	-0.16
Repair (% Change of Length) Dispersion	0.0010	0.25
Collagen Fiber Stiffness k_i_	0.031	-1.1
Soleus Maximum Isometric Force F_max_	-0.043	0.014
Soleus Maximum Shortening Velocity V_max_	-0.044	-0.12
Calcaneus Length L_calc_	0.066	0.36

## Discussion

The normal physiological processes occurring within tendon have been described in some detail. These processes enable the tendon to maintain its normal functional mechanical properties and to adapt to its local musculoskeletal environment when needed. However not a lot is known about the details of how tendon homeostasis and adaptation occurs [21, 26, 100, 101], and very little is known about how the basic processes of damage and repair are integrated to result in functionally relevant tendon responses.

Though simplified, the tendon model presented here is based on the normal physiological processes taking place in the Achilles tendon. We first combined known details of strain-mediated proteolytic and mechanical damage on collagen fibers into an integrated tendon-fiber damage and repair model. We then set the new tendon model within a simplified Achilles-soleus tendon model. Then in a very simple way (i.e. via our ‘beta function’), we set the musculotendon unit within the whole organism. Finally, we examined how the tendon remodels when perturbed from its equilibrium state. We demonstrated that for suitable model parameter selection (i.e. those parameter selected as described during model development), our tendon model is able to remodel the tendon in such a way that it approaches an equilibrium geometry, which is stable. Because of the way the beta function is formulated, this equilibrium tendon geometry also coincides with the minimum metabolic cost of generating force and mechanical work by the musculotendon unit.

How does tendon remodeling occur? The two tissue damage processes (mechanical damage and proteolytic damage) and their interaction within the context of the musculotendon unit operation, are fundamental to understanding the tendon adaptation model presented here. The basic properties of the mechanical and proteolytic damage and repair models are demonstrated in Figs 8 and 9. Then we demonstrate the behavior of the model tendon set within musculotendon unit, set within the whole organism, as shown in Fig 12. Fig 12 demonstrates regions of tendon length stability and regions of tendon length instability.

How do we understand this tendon length behavior within the musculotendon unit, set within the whole organism? At tendon lengths where musculotendon unit metabolic cost is low, (i.e. where muscle efficiency and load intensity is largest), tendons undergo high rates of mechanical damage, while at very short or long tendon lengths where musculotendon metabolic cost is high, (i.e. where the muscle becomes less efficient and load intensity reduces), mechanical damage rate is reduced. The reverse is true for proteolytic damage (e.g. where muscle efficiency is largest, proteolytic damage is lowest).

**Fig 13:**
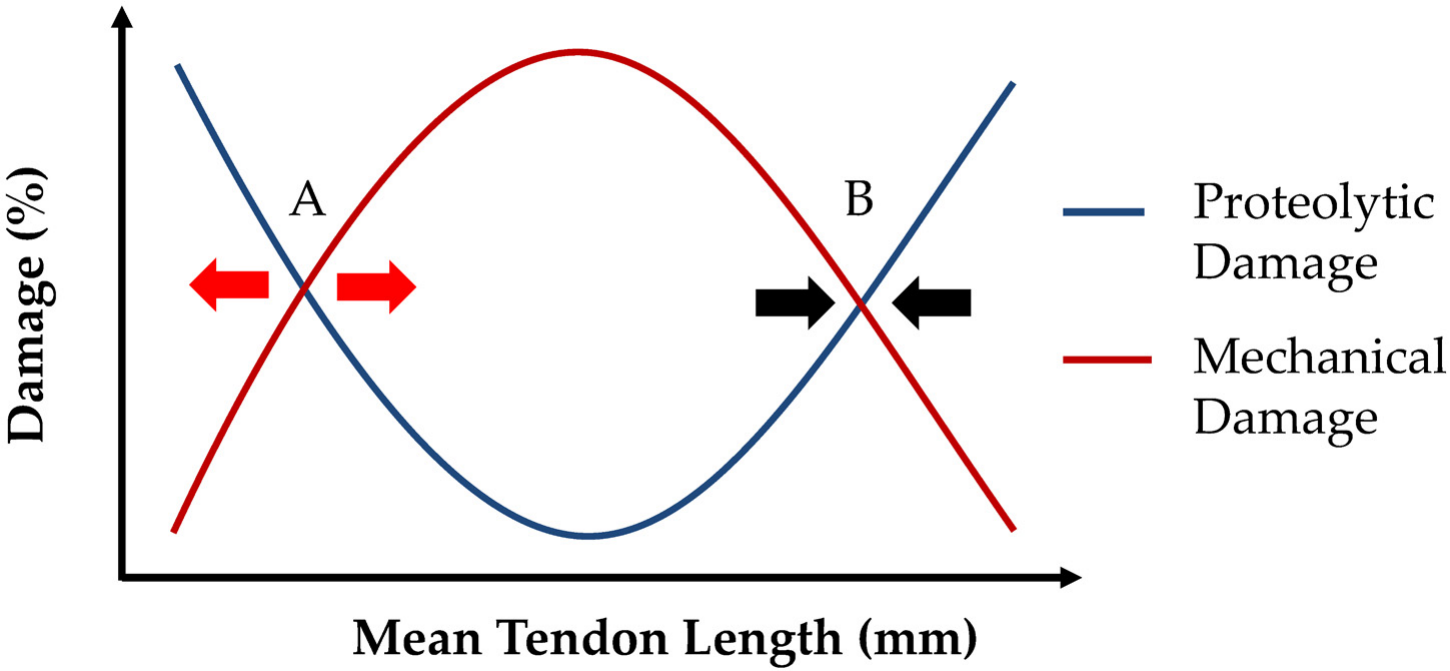
Relative relation of mechanical and proteolytic damages at different mean tendon lengths. Intersection at point A denotes an unstable state where remodeling lengths diverge. Point B denotes a stable state where remodeling lengths converge. Arrows signify the direction of tendon length change driven by damage and repair processes.

If we now assume that any damage is repaired by the tendon repair processes as described above, then the qualitative structure of these damage relationships shown in Fig 13 define regions of tendon length stability. For when the rate of mechanical damage is greater than the rate of proteolytic damage, the tendon lengthens. In contrast, where the proteolytic damage is greater than the rate of mechanical damage, the tendon shortens. This implies Point A in Fig 13 is an unstable point with respect to tendon length, i.e. following a geometrical perturbation from Point A, the tendon must either lengthen or shorten (in other words, its length diverges from A), while Point B is a stable point with respect to tendon length, that is, following a perturbation from Point B, the tendon length will always return to Point B. This stability structure of the tendon model set within the musculotendon unit, set within the whole organism, explains our model findings shown in Fig 12. In Fig 12, the lighter shaded points represent tendon lengths to the left of point “A” in Fig 13 (i.e. the lighter shaded points are in the domain of divergent initial states), while the darker points correspond with the tendon lengths to the right of point “A” (i.e. the darker shaded points are in the domain of convergent initial states). The domain of convergent initial states remodel towards the (attractor) equilibrium state (i.e. point “B” in Fig 13).

In it important to realise though, that a tendon at a stable geometrical configuration is in a *dynamic* equilibrium state. In other words at equilibrium, tendon damage and repair continue even though the tendon length (and fiber dispersion) are not changing (see Fig 11). In essence, when at homeostatic equilibrium, the tendency for the tendon to reduce its length is exactly balanced by the tendency for the tendon to increase its length. We see that anything upsetting this dynamic balance will lead to a change in tendon length. So for example, if the mechanical damage curve in Fig 13 is ‘pulled down’ (e.g. exercise level is reduced) while the rate of proteolytic damage is held constant, the tendon will shorten to find a new equilibrium state. On the other hand, if the rate of proteolytic damage is decreased (e.g. collagenases are inhibited) while the rate of mechanical damage remains constant, the tendon will lengthen to find a new equilibrium state. As long as this model structure is maintained (e.g. the two damage curves always intersect), this suggests that the model will operate successfully over a relatively wide range of parameter values.

This is confirmed by the sensitivity analysis shown in Table 2. For example, the sensitivity analysis shows that any tendon model parameter changed by 10% will lead to a change in tendon length of less than 1%. In other words, substantial changes in tendon model parameters are required to cause significant changes in tendon length. This may help explain why large changes in equilibrium tendon length are not commonly observed *in vivo*—in addition to requiring considerable time to reach a new equilibrium, changes in equilibrium tendon length would require significant changes in model parameters.

On the other hand, Table 2 shows that our model fiber length dispersion is about an order of magnitude more sensitive to parameter changes than its sensitivity to tendon length changes. Interestingly, this suggests that changes in tendon performance *in vivo*, may be reflected first by changes in fiber dispersion, which would alter tendon stiffness (for example tendon stiffness is likely to increase as fiber dispersion decreases).

In the tendon fiber model described above, we found that if the standard deviation of the collagen fiber lengths is initially greater than that of an equilibrium state, then the remodeling process results in a stiffening of a tendon (Fig 7), much like the stiffening reported in the literature [102]. This suggests that changes in the musculotendon unit associated with exercise may result from a decrease in fiber length dispersion. While we do not pursue this hypothesis in this paper, this brings us to discuss the structure of the probabilistic repair functions.

To simplify the model, we have assumed the repair function for mechanical fatigue damage to be the mirror image of the repair function for proteolytic damage. In a more general formulation of the model, there is no reason to suppose this be the case (e.g. as when an animal is growing). It is also apparent that the asymmetric structure of both lengthening and shortening employed in repair functions has an important influence on the rate at which an equilibrium state is achieved, and on the collagen fiber distribution at equilibrium. We found that if the variance of the repair functions is kept constant, while the expected values for fiber length adjustments is increased, i.e. the mean values of the fiber distributions shown in Fig 4(b) and 4(d) are increased, then the speed of convergence to an equilibrium state is increased. In other words, the speed of progression along the paths A-D in Fig 10 can be adjusted through the repair functions, however the equilibrium tendon length is found to relatively invariant to change in mean repair length (see Table 2).

It is clearly apparent that damage functions (see Fig 13) themselves depend on environmental conditions experienced by the tendon. It is also clear that tenocytes in the Achilles tendon are well placed to perceive any change in environmental conditions, and to adjust their secretion profile accordingly. It seems likely the tenocytes detect both strain and strain rates, which they ‘interpret’ by changing their local secretion profile [103]. A change in the secretion profile will influence both the proteolytic degradation profile and the repair functions, but these relationships remain to be elucidated experimentally.

In our model, an increase in dispersion of fiber lengths not only leads to a more compliant tendon, but also accelerates fiber damage under identical levels of activity. With continued increase in dispersion of the fiber distribution, eventually the rate of fiber damage may possibly exceed the capacity of the tendon to repair itself. In terms of our model, such an imbalance between damage and repair processes may help potentially explain disease states such as tendinosis and tendon rupture. It is possible, indeed likely, that the mean and variance of the repair functions depends on a variety of environmental factors including strain, strain rate, activity level, hormonal and nutritional states, as well as a variety of genetically and epigenetically determined factors. Unfortunately little or no quantitative experimental data about such relationships exist, and so clearly a large number of questions remain to be answered.

Despite its importance to muscle performance, there is little published information to suggest that tendon slack length changes with exercise. This is possibly due to the multiple shortcomings of published research on tendon adaptation studies to date [8, 9]. Nevertheless it is clear that tendons do change their length in both animal models and humans. For example, it has been demonstrated in a growing rabbit that tendon reattachment resulted in tendon lengthening accounting for approximately half of total length change of the musculotendon unit [104]. Indeed, the tendon itself experienced an increase in its initial length of about 15%, which demonstrates that the tendon incorporated new collagen fiber at an exceptionally rapid rate over an 8 week period.

There is also clear evidence of gastrocnemius tendon length adaptation in adult Guinea Fowl from a running exercise experiment (the treatment group had 30 to 60 minutes exercise 5 days per week for minimum or 8 weeks), while control animals remained caged) [7]. It is reported that for the treatment group gastrocnemius tendon stiffness increased 100%, while the gastrocnemius tendon lengthened by 25% [7].

Here we are particularly interested in what happens in adult human Achilles tendon. Surgical repair of ruptured Achilles tendon provides an opportunity to gain some insight into the capacity of Achilles tendon to remodel itself, but first we need to acknowledge that ruptured Achilles tendon must be in an abnormal state initially, and so how a ruptured tendon responds following surgical repair needs to be interpreted cautiously. The response may have less relevance to normal tendon physiology, as for example, inflammatory processes are involved in tendon remodeling for at least the first several months [22]. Nevertheless, closely monitoring the post-surgical changes in Achilles tendon demonstrates that over a twelve months period post-surgically the free Achilles tendon significantly changed its length, becoming up to 10 mm shorter or longer [105].

But perhaps the best *in vivo* evidence for adult human tendon length adaptation is evidenced following knee arthroplasty. Davies et al [106] provides data reporting substantial patellar tendon length adaptation over five year periods following knee arthroplasty. After examining 50 patients (n = 150 procedures), Davies et al [106] reports that following total knee arthroplasty, 38% of patellar tendons shortened by at least 10% after 5 years, while uni-compartment knee arthroplasty resulted in patellar tendon lengthening by at least 10% in 24% of patients after one year, and 22% after five years. Multiple additional studies on patellar tendon lengthening and shortening following knee replacement clearly demonstrate the capacity of adult human patellar tendon to remodel its length [107–109].

For habitual repetitive activities such as walking, energy minimization is regarded by some as a key optimization criterion dictating locomotor behavior [89, 90]. There is much evidence pointing to movement patterns that minimize energy expenditure, from the selection of preferred walking speeds in humans and other species [91, 92] to preferred stride frequencies [93, 94] and preferred gaits [95, 96]. Here we have shown that following a geometric perturbation in tendon geometry, our model tendon geometry can remodel to its equilibrium state in a biologically plausible way, which can coincide with minimizing the metabolic cost of the musculotendon unit. This finding corroborates that of Lichtwark and Wilson, who found optimal Achilles tendon properties exist for minimizing muscular energetic costs [110].

Although unintended in the sense it was not originally a goal of the tendon model developed here, the model also provide valuable insights and a possible explanation into a puzzling problem reported in the experimental literature about tendon, cartilage and bone. It has been experimentally established that the rate of collagen fiber turnover is remarkably slow while the metabolic activity of tendon is remarkably high [111]. That the metabolic activity of tendon is high is evidenced by the rate of procollagen synthesis per day in tendon is found experimentally to be around 1% of the total collagen mass (meaning the whole tendon could be replaced in a matter of about 3 months) [112, 113]. Yet, in contrast to the rapid rates of collagen turnover noted above, carbon 14 measurements indicate a ‘very limited’ rate of adult collagen fiber turnover [114]. The very slow rate of collagen fiber turnover and the seemingly contradictory finding of very high procollagen synthesis rates have yet to be explained.

We first consider the rate of collagen fiber turnover. For the exercise level considered in our model (which has Achilles tendon experience 5,000 load cycles per day), the total collagen fiber turnover time at the equilibrium state is predicted to be about 180 years (Fig 11). If the activity levels in our model are reduced to those associated with a more usual urban lifestyle in a well-developed country (say 2,000 load cycles per day), our model estimates the collagen fiber turnover time at an equilibrium state would increase to around 270 years. Significantly, though our intention was not to examine collagen turnover rates, these estimates appear to be consistent with observations on collagen fiber turnover rates reported in the literature. For example, a turnover rate of 197 years is reported for tendon in skeletally mature horses [115], while Thorpe et al (2010) estimated similar tendon collagen turnover rates in humans[116], and Silvin et al. (2008) report a half-life in human intervertebral disc collagen of 95 to 215 years [117]. Finally Verzigl et al. report a half-life of collagen in human articular cartilage of 117 years [117]. We note these measurements are in general agreement with our model predictions for the half-life of Achilles collagen fiber turnover. As far as we are aware, no quantitative explanation for experimentally observed collagen fiber turnover rates has been previously offered.

Now turning to the high procollagen production rates observed in Achilles tendon, it is also clear that for non-equilibrium Achilles tendon length states in the model tendon (i.e. when the tendon is in the process of changing its length), collagen degradation and synthesis turnover times differ, sometimes quite dramatically, from the equilibrium, dynamic steady-state turnover rate estimates (Fig 11). For example, our tendon model predicts that when a ‘long tendon’ is rapidly shortening to its equilibrium state, the peak degradation turnover time may decrease to below 5 years (Fig 11(b)). This clearly indicates that under certain environmental conditions, very high rates of procollagen synthesis and matrix MMP expression may be required to facilitate very rapid tendon adaptation [118]. It seems plausible that large rates of procollagen production may be maintained just in case circumstances arise when rapid remodeling of the tendon is required, e.g. as depicted in Fig 11.

In any case we mention here that estimating collagen turnover played no part in our model calibration. In other words, the collagen fiber turnover predictions made by our model are independent estimates, yet they are remarkably consistent with turnover times reported in the experimental literature [114–117]. This outcome is very encouraging as it helps to build confidence in the model, while pointing to further utility of our proposed tendon model formulation.

### Conclusion

The tendon adaptation model presented here primarily revolves around the interplay between mechanical and proteolytic damage and repair processes operating in physiologically normal Achilles tendon. It is now well established that mechanical fatigue can damage collagen fibrils, that MMPs are present in tendon and they proteolytically degrade collagen fibrils, that mechanical strain reduces and can even prevents proteolytic degradation of collagen fibrils, and that collagen fibrils can be repaired *in vivo*. But what is not yet known is how these individual processes are functionally integrated *in vivo* to facilitate tendon homeostasis and adaptation [100, 101].

The key achievement in the present work is that we have demonstrated how these processes can be logically combined to facilitate tendon length adaptation in a robust fashion. In the content of our model assumptions, and for suitable parameter selection (which based on a sensitivity analysis appear to be robust), we show that the tendon can autonomously remodel until it reaches a stable, equilibrium length state. Assuming that the multi-objective function representing external influences on the musculotendon unit is dominated by musculotendon unit economy, we find the perturbations of tendon geometry result in remodeling towards a stable tendon geometry, which coincides with a region minimizing the metabolic cost of muscle activity. As with the initial development of any model, we have invoked many important model simplifications in the interests of building a parsimonious model to highlight fundamental theoretical concepts. But it is clear that upon relaxing these model simplifications, there is great scope for further subtleties of tendon adaptation to emerge. Building upon the foundation established here, it seems likely that more sophisticated and complex extensions of the model will reveal important new interactions that may help explain experimental observations or suggest new experiments on tendon biology.
